# New onset contralateral acute epidural hematoma following decompressive craniectomy for an acute subdural hematoma: A report of 2 cases

**DOI:** 10.1016/j.tcr.2025.101236

**Published:** 2025-08-21

**Authors:** Dawit Workneh Gechu, Mehari Wale Alem, Yordanos Girma Legesse

**Affiliations:** a Addis Ababa University, Ethiopia; b Aletha Medical Center, Ethiopia; c University of Gondar, Ethiopia; d St. Paul's Hospital Millennium Medical College, Ethiopia

**Keywords:** Acute extradural hematoma, Subdural hematoma, Decompressive craniectomy

## Abstract

**Background:**

Contralateral acute epidural hematoma development following a decompressive craniectomy for an acute subdural hematoma is a rare but fatal complication. Early recognition and treatment can be lifesaving.

**Observation:**

We present 2 cases of a new onset massive contralateral acute epidural hematoma development following decompressive craniectomy and hematoma evacuation for acute subdural hematoma, each with different course of the pathology and post-operative outcome.

**Lessons:**

Even after a decompressive craniectomy for a structural primary lesion, neurologic follow-up and immediate control CT scans of patients with severe traumatic brain injury (TBI) can help us identify this very uncommon complication. Additionally, these two cases demonstrate the significance of prompt detection and intervention, which can dramatically change the pathology's progression and neurologic outcome.

## Introduction

Traumatic brain injury progresses over time, resulting in the development of multiple lesions [1]. Isolated lesions account for only 30–40 % of Acute Subdural Hematomas (ASDH) that require surgery. Intracerebral hematomas and contusions are the most commonly reported intracranial abnormalities. Six to 14 % of patients have an associated epidural hematoma that is initially visible. In cases of acute subdural hematoma, where the brain is typically tense and swollen, it is rare for a delayed hematoma to develop after decompressive craniectomy. Acute epidural hematomas, acute subdural hematomas, and intra-parenchymal cerebral hematomas—rarely cerebellar—are among the various bleeding patterns that have been reported. The bleeding may be ipsilateral (the most common), contralateral, or, in rare cases, bilateral [2].

## Case presentation

### Case 1

#### History

This is a 32 years old, right handed male pedestrian who presented after sustaining a motor vehicle accident. He was referred from a local health center 4 h after the accident. At presentation he had decreased mentation, 1 episode of generalized tonic clonic seizure and right side body weakness.

#### Physical examination

He was acutely sick looking with a blood pressure of 140/80, Respiratory Rate of 28–32, pulse rate of 62–73, and an oxygen saturation of 85 % on ambient air. He had transmitted sound on the bilateral lung fields. He was comatose with Glasgow coma scale (GCS) of 7 (E1V1M5), moving only left side of the body. He was on cervical collar. Pupils were dilated and slowly reactive to light on the left side and mid-size and reactive on the right side.

#### Laboratory and imaging workups

**Fig. 1:**
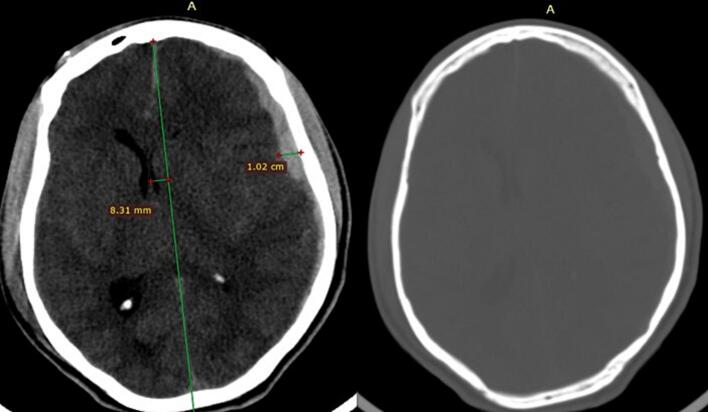
Lt. Side ASDH with maximal thickness of 1.02 cm and MLS of 8.31 mm, No contralateral hematoma or skull fracture on bone window.

He was immediately intubated and resuscitated according to ATLS protocol. Emergency Brain & C-spine CT scan was obtained and it showed a left side 1 cm thick holo-hemispheric ASDH with a midline shift (MLS) of 8.3 mm and effacement of basal cistern. There was no evidence of skull fracture on the bone window as well as the 3D reconstructed sequence (Fig. 1). Basic Laboratory evaluations (CBC, electrolytes, Coagulation profile, renal function test and Liver panel) didn't show any abnormalities (Fig. 1.

#### Management and course of the patient

**Fig. 2:**
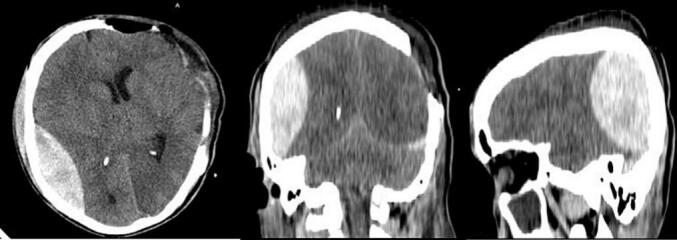
Post decompressive craniectomy changes with contralateral right side P-O AEDH with maximal diameter of 2.91 cm and 1.5 cm MLS to the left side.

After securing the airway and resuscitation, Mannitol was started and patient was taken directly to the OR. He underwent a left side decompressive craniectomy, hematoma evacuation and expansile duraplasty. There was no evident intraoperative brain swelling. Patient was transferred to the ICU stable and put on sedation. Six hours after the surgery patient was clinically assessed and put off sedation. Chest was clear and neurologic evaluation showed GCS of 10 T (E4VTM6). He was extubated next morning and put on face mask O2 support. Four hours after the extubation his GCS dropped to 9 (E2V2M5) and contralateral pupil was dilated and NR to light. He was re-intubated and urgent CT scan was obtained. It showed a contralateral P—O AEDH with mass effect and MLS to the Left side (Fig. 2). He was immediately taken to the OR, a right side P—O craniotomy and Hematoma evacuation was done (Fig. 2.

#### Patient follow-up

Post operatively patient was kept in the ICU intubated and sedated for the next 2 days. He showed progressive neurologic improvement. On the 3rd day he was extubated and transferred to the wards later on. At discharge he was conscious and oriented with mild residual right side weakness. On subsequent follow-ups he was ambulatory and takes care of himself. 3rd month Glasgow outcome score (GOS) was 6. Cranioplasty with autologous bone was done at 6 month post-op.

### Case 2

#### History

A 21 years old male patient came in with loss of consciousness after falling from roof of a car on to the ground 4 h back. At presentation he was unresponsive and had 2 episodes of projectile vomiting. He also had fast and labored breathing.

#### Physical examination

**Fig. 3:**
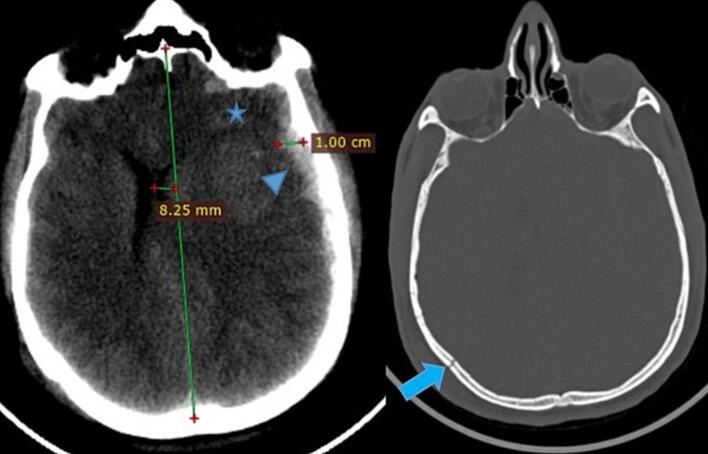
Left side ASDH (arrow head) with diameter of 1cm and MLS, multiple contusions (star) of frontal lobe and Rt. Side temporal linear fracture (arrow).

He was acutely sick looking with respiratory distress. His vital signs were blood pressure of 160/90, respiratory rate of 35, pulse rate of 68, Temperature of 37.1 and an oxygen saturation of 74 %. Chest was full of transmitted sounds on bilateral lung fields. On neurologic examination, his post resuscitation GCS was 7/15 (E1V1M5), Pupil were mid-size bilaterally with left side slowly reactive to light and Right one reacting normally. He had left side motor preference but moves all extremities (Fig. 3.

#### Laboratory and imaging

Basic laboratory investigations including coagulation profile were normal. *E*-fast and trauma series X-ray were done, results were unremarkable. Head and C-Spine CT were obtained and showed left side acute subdural hematoma with bilateral Frontal punctate hemorrhagic contusions. There was also a right side Parieto-Temporal linear skull fracture (Fig. 3).

#### Management and course of the patient

**Fig. 4:**
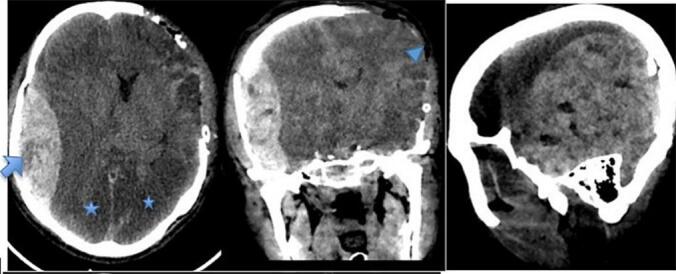
Right parieto-temporal AEDH with swirl sign (arrow) causing MLS and bilateral PCA territory infraction (star) and craniectomy defect (arrow head).

The patient was immediately intubated in the ER and put on mechanical ventilator. He was taken directly to the OR, underwent left side decompressive craniectomy, hematoma evacuation and expansile duraplasty. An intraoperative progressive swelling of brain parenchyma was noticed. Closure was done fast and patient was transferred to the ICU. Control CT scan was obtained, which showed massive contralateral P-T acute epidural hematoma (AEDH) with Max diameter of 3 cm causing MLS to the left side of 1.7 cm with uncal and tonsilar herniation. There was also bilateral PCA and focal MCA territory infraction (Fig. 4). Patient was also not wakeful post-op, pupil were dilated and slowly reactive on the right side and left side was mid-size and slowly reactive to light. He was taken back to OR after 6 h in the ICU from the initial surgery. Right Parieto-Temporal Craniotomy and hematoma evacuation was done. The patient was transferred back to the ICU and put on mechanical ventilator. Following the 2nd surgery the patient had a stable course but showed no neurologic improvement. Pupils remained dilated and non-reactive. On the Subsequent days brainstem reflexes were absent and apnea test confirmed brain death. He had cardiorespiratory arrest on 5th post-operative day (Fig. 4.

## Discussion

According to published articles, surgical site herniation, post-operative infections, epilepsy, and subdural effusions with or without hydrocephalus are the most frequent surgical complications following a decompressive craniectomy for an acute subdural hematoma [1].

One well-known but dangerous side effect of intracranial procedures is post-operative delayed hemorrhage, which typically happens at the operation site. Rarely does a new onset intracranial hemorrhage occur far from the craniotomy site; instead, intracerebral hematomas occur in the majority of cases [3].

The mechanism of delayed contralateral AEDH has been explained by a number of theories. The loss of the tamponade effect on the bleeding source is one of them. The development of the EDH would be prevented until a decompressive craniectomy was performed on the contralateral side, which would lower the intracranial pressure and relieve the hemostatic tamponade, allowing the development of the epidural hematoma. The mass effect from the countercoup acute subdural hematoma and contusions most likely increases the intracranial pressure and produces a tamponade effect on the contralateral EDH. Additional hypothesized mechanisms include acute coagulopathy, aberrant vasomotor mechanisms, aggressive anti-edema measures, and iatrogenically induced (during initial surgery) [1,3].

Pupil asymmetry, delayed post-operative neurologic deterioration, and intraoperative brain swelling were observed in the majority of documented cases. Persistently high ICP in patients with ICP monitors may also be a sign of post-operative delayed EDH collection [1,4]. Imaging signs for new onset or expanding delayed AEDH include thin film of epidural or subdural hematoma, skull fracture, contusions, and pneumocephalus [4].

MMA branches and dural venous sinuses—the latter of which presents more slowly—are the sources of the bleeding that have been identified in the literature [4,5]. Extending the CT angiography study, which is commonly done during poly-trauma evaluations, to include the brain is one possible way to detect an imminent contralateral EDH. This might make it easier to find an MMA tear leak [4]. In order to treat these cases, an emergency craniotomy and hematoma evacuation were required. Exploratory burr holes may still be useful in treating the lesion and averting death in cases of intraoperative brain swelling, whether or not preoperatively determined risk factors for delayed suspected AEDH are present [1].

The loss of ASDH's tamponade effect after DC is most likely the cause of the delayed epidural hematoma in both of our patients, and the MMA-avulsed branches were found to be the source of the bleeding. The second case's delayed surgical intervention was probably the cause of the difference in postoperative outcome. Financial difficulties and the absence of a portable mechanical ventilator to transfer the patient to the CT unit were the reasons for the delay. Given the contralateral skull fracture and progressive intraoperative brain swelling seen on the initial brain CT scan, this patient may have also benefited from an exploratory burr hole.

## Conclusion

This case highlights the importance of close clinical monitoring of patients who have severe TBI, even after decompressive procedures in resource limited settings where ICP monitoring devises aren't available. Early Post-operative control CT scan after decompressive craniectomy are also crucial in detecting remote Post-operative extradural collections, especially in the setting of new onset neurologic deterioration and intraoperative brain swelling. The above 2 cases highlight, timely and efficient surgical intervention is of paramount importance in saving the lives of these patients.

## Abbreviation

AEDH: acute epidural hematoma
ASDH: acute subdural hematoma
DC: decompressive craniectomy
EDH: epidural hematoma
GCS: Glasgow Coma Scale
GOS: Glasgow Outcome Scale
ICP: intracranial pressure
MCA: middle cerebral artery
MLS: mid line shift
MMA: middle meningeal artery
PCS: posterior cerebral artery
TBI: traumatic brain injury

## CRediT authorship contribution statement

**Dawit Workneh:** Writing – review & editing, Writing – original draft, Data curation, Conceptualization. **Mehari Wale:** Writing – review & editing. **Yordanos Girma Legesse:** Writing – review & editing.

## Ethical consideration

Written informed consent for publication of their clinical details and/or clinical images was obtained from the patients.

## Funding

None.

## Declaration of competing interest

The authors have no conflicts of interest to declare.
